# Unilateral Trigeminal Sensory Neuropathy with Sjögren’s Syndrome with Liver and Renal Impairment

**DOI:** 10.3390/neurolint13030045

**Published:** 2021-09-08

**Authors:** Kana Ozasa, Noboru Noma, Jumi Nakata, Yoshiki Imamura

**Affiliations:** 1 Department of Oral Diagnostic Sciences, Nihon University School of Dentistry, Tokyo 101-8310, Japan; deka18006@g.nihon-u.ac.jp (K.O.); imamura.yoshiki@nihon-u.ac.jp (Y.I.); 2 Clinical Research Division, Dental Research Institute, Nihon University, Tokyo 101-8310, Japan; 3 Division of International Medicine, Towa Hospital, Tokyo 120-0003, Japan; jumi.nakata@gmail.com

**Keywords:** quantitative sensory testing, Sjögren’s syndrome, anti-Ro/SSA, primary biliary cirrhosis

## Abstract

Liver and renal involvement is a rare event in Sjögren’s syndrome. Sjögren’s syndrome is characterized by the progressive immune-mediated destruction of epithelial tissues of the salivary and lacrimal glands. Sensory ganglionitis, accompanied by T-cell invasion, occurs in patients with Sjögren’s syndrome, resulting in sensory neuropathy of the face or limbs. Patients are assessed by quantitative sensory testing. A 76-year-old woman presented with numbness of her left face and was subsequently diagnosed with Sjögren’s syndrome and primary biliary cirrhosis, and was found to have renal failure. Detection of her serum anti-Ro/SSA antibody was strongly positive. Shirmer’s test or a salivary volume in the gum test also showed positive results. Her somatosensory disturbance severity was higher in the trigeminal area than in the forearm, suggesting that the trigeminal nerve is more susceptible than other parts of the nervous system in patients with Sjögren’s syndrome and primary biliary cirrhosis. A simple sensory test could be performed during regular check-ups, as sensory deficits might develop after patients are diagnosed with Sjögren’s syndrome and primary biliary cirrhosis.

## 1. Introduction

Peripheral neuropathy has been reported to be associated with Sjögren’s syndrome, which has been described as involving facial paralysis and trigeminal sensory impairment as a comorbidity [1]. Initially, the main cause of neuropathy was thought to be vasculitis [2]. However, Malinow et al. demonstrated that inflammatory cell infiltration into the dorsal root ganglion induces sensory ataxia-type neuropathy, suggesting that the target of pathological manifestation is the ganglia rather than vasculitis [3].

Peripheral neuropathy, most commonly the distal sensory symmetrical type, is present in 10–20% of patients with primary Sjögren’s syndrome [4]. Isolated cranial nerve lesions in primary Sjögren’s syndrome, affecting the third through tenth nerves, have been reported previously [1,5,6,7,8,9]. In addition, pure sensory neuropathy of the limbs with trigeminal nerve involvement was described in patients with Sjögren’s syndrome [2]. In general, cranial nerve involvement is typically bilateral in patients with Sjogren’s syndrome. It has been reported that hyperalgesia, allodynia, hypoesthesia, and hypoalgesia in the orofacial region precede Sjogren’s syndrome [9]. Somatosensory features might indicate clinically painful (gain-of-function; hyperalgesia, allodynia) or not painful (loss-of-function; hypoesthesia) signs of trigeminal nerve dysfunction. However, the results of a quantitative sensory test (QST) protocol for the assessment of trigeminal sensory neuropathy due to Sjögren’s syndrome have not been reported to date. Here, we report a rare case of Sjögren’s syndrome in a patient presenting with unilateral trigeminal sensory neuropathy and primary bile cirrhosis.

## 2. Case

**Figure 1:**
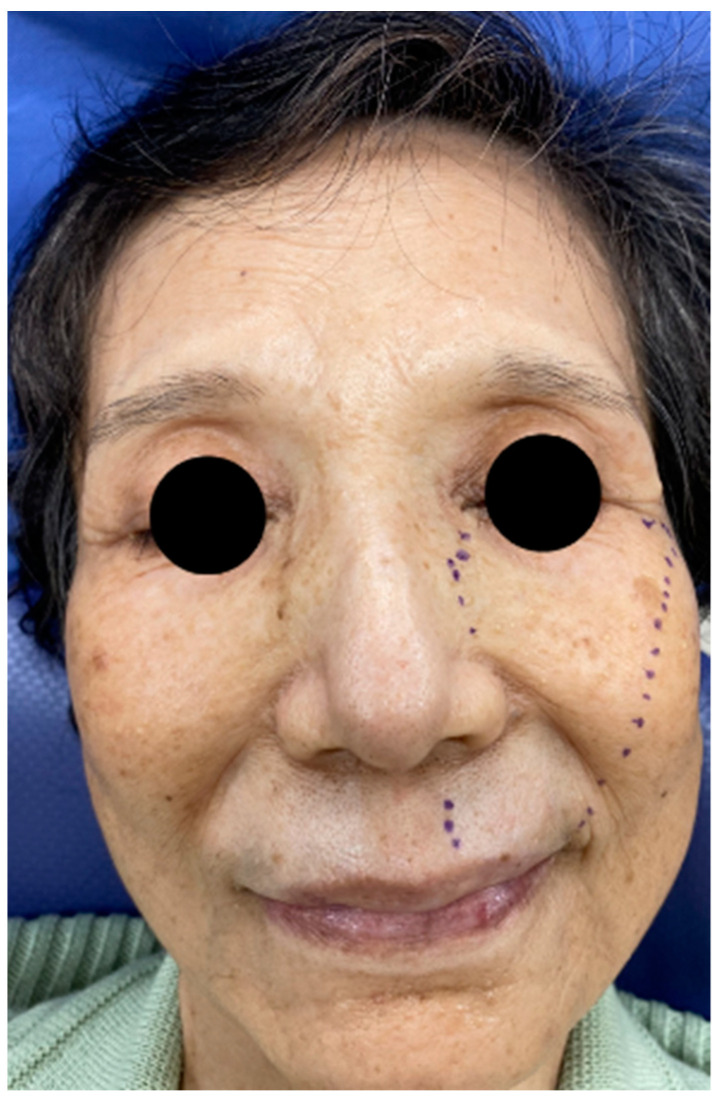
Range of hypoesthesia on the left face of the patient.

The patient was a 76-year-old woman. She had left upper lip numbness, dry eyes, and dry mouth 5 months prior. The numbness gradually spread to the infraorbital area (Figure 1). She had no painful sensation, but often chews on the left buccal mucosa because of numbness. She was diagnosed with primary biliary cirrhosis based on a liver biopsy and positive antimitochondrial antibody 20 years prior. At the same time, further laboratory tests showed positive anti-SSA and anti-SSB antibodies, and a minor salivary gland biopsy resulted in a diagnosis of secondary Sjögren’s syndrome. The patient was treated with prednisone (10 mg) orally daily for a few years, and after that, ursodeoxycholic acid was administered to treat primary biliary cirrhosis. Approximately 4 months before seeking treatment, the patient was aware of diminished visual acuity due to glaucoma; optic neuritis was ruled out by an ophthalmologist. Magnetic resonance imaging was performed at the Department of Neurology of Juntendo University Hospital, and patients with multiple sclerosis or central neuropathy were excluded.

Simple neurosensory testing results showed decreased perception of a pinprick light touch and temperature at the second branch of the left trigeminal division (V2), including the intraoral area. Cranial motor function and the masseter reflex were intact. The remaining neurosensory status was normal.

Laboratory investigations revealed a hemoglobin level of 10.5 g/dL, red blood cell count of 329 (10^6^/μL), white blood cell count of 4.1 K/mcl, erythrocyte sedimentation rate of 42 mm/h, platelet count of 333 K/mcl, Lymp (20%), MCV (91.0 u^3^), MCH (31.3 pg), and MCHC (34.4%). Renal panel and liver function tests showed the following results: blood urea nitrogen of 23, creatinine of 0.86, estimated glomerular filtration rate of 49, γ-GTP of 66, AST of 51, alanine aminotransferase of 26, and lactate dehydrogenase of 165. Complement component 3 and 4 levels were normal. Immunological tests showed a C-reactive protein level of 0.1 mg/dL, antinuclear antibody (160), IgM (160), and IgG (1682). Serum anti-Ro/SSA antibody was strongly positive for borderline anti-La/SSB. The Shirmer test was positive (right: 5 mm, left: 7 mm). The salivary volume in the gum test was 0 mL/10 min, and lip biopsy revealed extensive lymphocytic infiltration with fibrotic changes in the salivary gland.

### 2.1. CT X-ray-Findings

**Figure 2:**
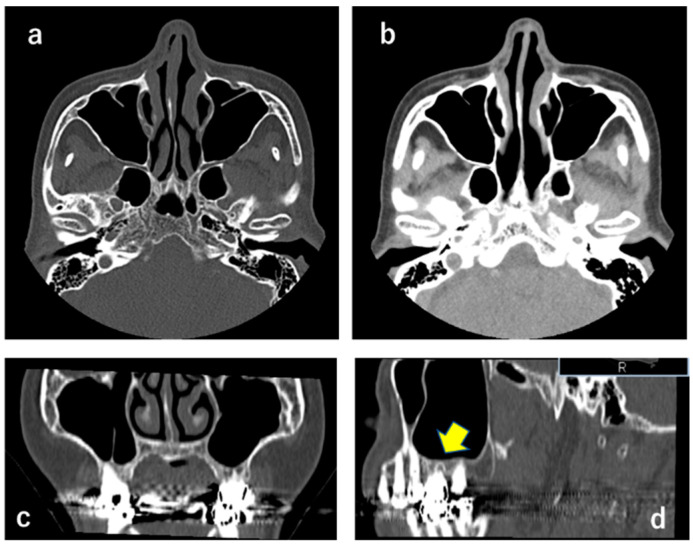
CT X-ray showing that masses were not observed in the left maxillary sinus (**a**–**c**). (**b**) indicates soft tissue density. A sagittal section ((**d**); yellow arrow) shows an apical periodontitis-like image at the maxillary left first molar, suggesting that this was not relevant to the clinical findings.

Abnormal findings such as a mass or alveolar bone resorption were not observed in the left maxillary sinus, suggesting that the left infraorbital nerve was not compressed (Figure 2).

### 2.2. QST

The comprehensive standardized QST protocol of the German Research Network on Neuropathic Pain (DFNS) was performed at the second branch of the left trigeminal division or left forearm [10]. The QST protocol of the German Research Network on Neuropathic Pain was performed [3]. Abnormal patient QST parameters showed the thermal sensory limen (TSL = 32.6 °C), cold pain threshold (CPT = 0 °C), mechanical pain threshold (MDT = 1.63 g), and pressure pain threshold (PPT = 1.2 kPa). Of these, TSL, CPT, MDT, and PPT at the trigeminal region indicated a loss of function. In contrast, all parameters were within the normal limits in the forearm.

### 2.3. Diagnosis

From the perspective of facial numbness, a differential diagnosis includes herpes zoster, HIV infection, osteomyelitis, neurological diseases such as multiple sclerosis, malignant lymphoma, cerebral infarction, space-occupying lesions (acoustic nerve tumors, epithelioma), jawbone tumors, megaloblastic anemia, and metastatic tumors. Neurologists ruled out central nervous neuropathy using MRI and other methods, and we ruled out peripheral nerve neuropathy. Based on QST testing, thermal and mechanical QST showed elevations of TSL, CPT, MDT, and PPT, and hypoalgesia was marked, suggesting not-painful trigeminal neuropathy. The diagnosis of possible unilateral trigeminal sensory neuropathy with Sjögren’s syndrome was made.

## 3. Discussion

Based on the International Classification of Orofacial Pain, oral mucosal pain attributed to autoimmunity includes Sjögren’s syndrome, lupus erythematosus (systemic or discoid type), and erythema migrants [10]. Sjögren’s syndrome is a systemic autoimmune disease that frequently presents concomitantly with other systemic connective tissue or organ-specific autoimmune diseases. This association has been extensively described in systemic lupus erythematosus and rheumatoid arthritis. Oral mucosal tissues can become abraded and even cut by dry foods and sores [10]. In our case, the patient complained of mild pain on the superficial mucosa due to dry mouth, but more often complained of pain caused by a bite to the left buccal mucosa because of numbness.

Trigeminal sensory neuropathy is characterized by slowly progressing bi-lateral facial numbness and occasionally paresthesia. Previous studies showed that, in most patients, neuropathy develops first, whereas Sjögren’s syndrome was diagnosed up to 1–12 years later [8]. In contrast, sensory impairment might appear as the first symptom of Sjögren’s syndrome [11]. Our case was considered rare because numbness appeared in the left face 20 years after the Sjögren’s syndrome diagnosis.

To the best of our knowledge, this is the first report of a comprehensive somatosensory profile of a patient with unilateral trigeminal sensory neuropathy with Sjögren’s syndrome. Patients with Sjögren’s syndrome show an extensive increase in the MDT (1.63 g), TSL (32.6 °C), CPT (0 °C), and PPT (1.2 kPa) on the affected side. Mori et al. reported that large sensory ganglion neurons are predominantly diminished in patients with Sjögren’s syndrome. Myelinated fiber density in the dorsal spinal segments was determined to be 48% of the control value in the C5, 42% in the Th11, and 22% in the L4 segments [12]. In the trigeminal nerve region, myelinated large fibers can be preferentially depleted over small fibers in patients with Sjögren’s syndrome. With respect to the pathological basis of the Sjögren’s syndrome-associated neuropathy, autopsy findings from a patient with the sensory ataxic form suggested that there is a continuous spectrum of pathological processes among the different forms of neuropathy. Sensory ganglionitis accompanied by T-cell invasion is also present in patients with Sjögren’s syndrome [12]. Urban et al. also observed ganglionitis with lymphocytic T-cell infiltration and sensory neuropathy of the limbs [8]. All forearm parameters in this study were within the normal range, supporting the notion that the trigeminal nerve is more susceptible in patients with Sjögren’s syndrome [13].

The exact frequency of Sjögren’s syndrome and renal impairment is unclear, but kidney disease occurs in 5% of patients with primary Sjögren’s syndrome [14]. There are many patterns of tubular interstitial nephritis. In addition, it also causes renal tubular acidosis, resulting in a decrease in the urinary concentration. Symptoms vary from asymptomatic to electrolyte imbalances, kidney stones, renal insufficiency, and nephrotic syndrome. In our case, renal panel function testing showed an eGFR of 49, but the patient was asymptomatic.

## 4. Conclusions

We report a rare case of Sjögren’s syndrome, with liver and renal involvement. Trigeminal sensory impairment in the left face developed 20 years after our patient was diagnosed with Sjögren’s syndrome; therefore, simple sensory testing might be helpful in identifying trigeminal neuropathy during regular checkups of suspected patients, especially when complaining of abnormal sensations.
